# Patterns of Urinary Incontinence Among Women in Asir Region, Saudi Arabia

**DOI:** 10.7759/cureus.21628

**Published:** 2022-01-26

**Authors:** Sultan Z Alshehri, Amjad K Abumilha, Khaled A Amer, Abdulrahman A Aldosari, Rammas A Shawkhan, Khalid A Alasmari, Tameem A Sabrah

**Affiliations:** 1 Urology, Aseer Central Hospital, Abha, SAU; 2 College of Medicine, King Khalid University, Abha, SAU

**Keywords:** saudi arabia, risk factors, prevalence, females, urinary incontinence

## Abstract

Background

Urinary incontinence among women is a widespread clinical condition that is frequently associated with marked physical, social, and psychological adverse impact that significantly worsens their quality of life. This study is conducted to identify the prevalence of urinary incontinence and associated risk factors among Saudi women in Asir Region, Saudi Arabia.

Methods

Following a cross-sectional design, 1,964 healthy non-pregnant females aged above 13 years attending primary healthcare centers in Asir Region, Saudi Arabia were included. A self-administered semi-structured questionnaire was used, which included sociodemographic and clinical characteristics, questions related to voiding habits, and the validated Arabic version of the International Consultation on Incontinence Questionnaire (ICIQ).

Results

Almost half of the participants (47.5%) had urinary incontinence, of which 26.8% was slight, 16.3% was moderate, and 4.3% was severe. About 15.2% used to leak urine once a week or less, 3.6% used to leak two to three times a week, and 5.6% leaked daily. The leaked amount of urine was small in 26% of participants, while it was moderate and large in 8% and 1.4% of participants, respectively. Leaking urine moderately affected the daily life of 21.9% of participants, while it severely affected 14.7% of participants. Their grades of urinary incontinence differed significantly according to their age group, with the highest prevalence of severe incontinence among those aged 50 years or more (p < 0.001). Grades of urinary incontinence also differed significantly according to participants’ marital status, with those married or widowed having the highest prevalence of severe incontinence (8.5% and 19%, respectively; p < 0.001). Moreover, its grades differed significantly according to participants’ number of pregnancies, with severe incontinence being highest among those with twice gravidity or three times or more (8.3% and 7.9%, respectively; p = 0.004). Grades of urinary incontinence also differed significantly according to the presence of associated diabetes mellitus and renal/urinary tract diseases (p = 0.001 for both).

Conclusions

Urinary incontinence is common among Saudi females. Older age, multiparty, and menopause are significant risk factors for urinary incontinence.

## Introduction

Urinary incontinence is a widespread clinical condition, especially among women [1]. It is the observation of involuntary leakage from the urethra, synchronous with effort or physical exertion or on sneezing or coughing [2]. It is frequently associated with the marked physical, social, and psychological adverse impact that significantly worsens women's quality of life [3], restricting their social activity, and is usually accompanied by medical complications. Though urinary incontinence is not life-threatening, it is a very debilitating condition. One woman was so tortured by urinary incontinence that she founded the Simon Foundation for Continence and became a spokesperson, appearing on television and radio programs throughout the world [4]. In the USA, its impact among females has been shown to cause a considerable financial burden, even exceeding the costs of breast cancer [5]. Moreover, the prevalence of urinary incontinence among American females has been reported to range from 8.5% to 58.0% [6]. About one-third of women after 40 years of age experience urinary incontinence [7]. The reported prevalence of urinary incontinence may range from 5% to 70% [8], due to differences in definitions, research designs, data collection tools used, study sample, and response rates [9,10]. In the Middle East, the overall prevalence of urinary incontinence ranges from 20.3% to 54.8%. In Qatar, it is 20.6%, and in the United Arab Emirates, it is 20.3% [11,12]. In Saudi Arabia, the prevalence of urinary continence in Jeddah and Riyadh cities was estimated to be 41.4% and 29%, respectively [6,13]. There are several risk factors associated with urinary incontinence, such as age, obesity, medical comorbidities, hysterectomy, and multiparity. Moreover, repeated pregnancies and deliveries may constitute a major risk factor among young and middle-aged women [14]. In addition, increased reporting of urinary incontinence among women is attributed to population aging and raised public awareness that urinary incontinence can be managed and it is not an acceptable part of normal aging [15]. Differences in risk factors predisposing women to urinary incontinence were reported in different communities [16,17]. Hence, the identification of predictors of urinary incontinence for the avoidance of risk factors involved is essential in preventing urinary incontinence [18], which will help to limit the negative impact of incontinence on quality of life and social activities that are common among women with incontinence [19].

The present study aimed to identify the prevalence of urinary incontinence and associated risk factors among Saudi women in Asir Region, Saudi Arabia.

## Materials and methods

Following a cross-sectional design, healthy non-pregnant females aged above 13 years attending primary healthcare centers in Asir Region, Saudi Arabia, seeking health care were invited to participate in the present study. Pregnant and postnatal women were excluded from the study.

The present study was approved by the Regional Committee for Research Ethics, Asir Region (approval number: REC-05-07-2021). After getting the informed consent, participants were asked to complete a self-administered semi-structured questionnaire, which included sociodemographic and clinical characteristics, questions related to voiding habits, and the validated Arabic version of the International Consultation on Incontinence Questionnaire (ICIQ) [20,21], which consisted of the following items: frequency of urinary incontinence (never, once a week, two to three times a week, at least once a day); volume (none, small amount, moderate amount, large amount); and "How much urine leakage affects your daily life?" (0: not at all; 1-3: mildly; 4-6: moderately; 7-9: severely; 10: to a great extent).

Using the following formula: n = z^2^.p.q/d^2^ [22], where Z statistics is 1.96, assumed prevalence is 25% for incontinence, and our desired accuracy is at 0.05 level, the minimum sample size was calculated to be 1,800 participants. However, the sample size was increased to 1,964 participants to compensate for missing data. The data were collected during the period from January 2021 to March 2021.

Urinary incontinence was considered as the primary outcome (dependent) variable, while risk factors were considered as the explanatory variables. Frequency and percentages were calculated for qualitative variables. The chi-square (c^2^) test was applied to test statistical significance. Data were analyzed using the Statistical Package for the Social Sciences (SPSS) version 25 (IBM Corp., Armonk, NY). P-values equal to or less than 0.05 were considered statistically significant.

Operational definition

Urinary incontinence is defined as involuntary leakage of urine among adult non-pregnant women [4].

## Results

Table 1 shows that the age of most participants (67%) was 18-29 years, while the age of 23.6% of participants was less than 18 years. Most participants were university graduates (66.6%). Most participants were single (87.5%), while 10.2% were married. The majority of participants (90.4%) had no previous pregnancies, while 7.1% had three or more previous pregnancies. Regarding associated comorbidities, 10.5% of participants were diabetic, while 20.4% had urinary tract diseases.

**Table 1:** Personal characteristics of the study sample

Personal characteristics	No.	%
Age group		
<18 years	463	23.6
18-29 years	1,315	67.0
30-39 years	84	4.3
40-49 years	80	4.1
50+ years	22	1.1
Educational level		
Primary/intermediate	47	2.4
Secondary	608	31.0
University	1,309	66.6
Marital status		
Single	1,718	87.5
Married	201	10.2
Widow	21	1.1
Divorced	24	1.2
Number of pregnancies		
0	1,775	90.4
1	26	1.3
2	24	1.2
3+	139	7.1
Present history of diabetes	207	10.5
Presence of urinary tract diseases	400	20.4

**Figure 1:**
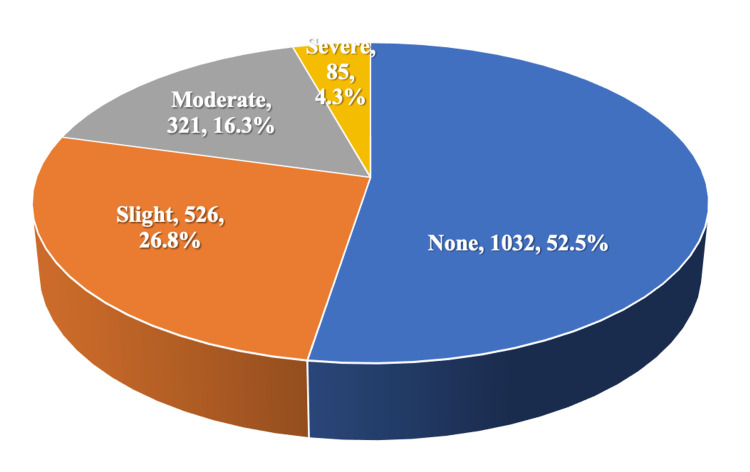
Grades of urinary incontinence

Table 2 shows that 15.2% of participants used to leak urine once a week or less, 3.6% used to leak two to three times a week, and 5.6% leaked daily. The leaked amount of urine was small in 26% of participants, while it was moderate or large in 8% and 1.4% of participants, respectively. Leaking urine moderately affected the daily life of 21.9% of participants, while it severely affected 14.7% of participants. Almost one-half of participants (47.5%) had urinary incontinence, of which 26.8% was slight, 16.3% was moderate, and 4.3% was severe, as shown in Figure 1.

**Table 2:** Patterns of incontinence among participants

Patterns of urinary incontinence	No.	%
How often do you leak urine?		
Never	1,486	75.7
Once or more a week or less	299	15.2
2-3 times a week	70	3.6
Daily	109	5.6
How much urine do you usually leak?		
Nil	1,268	64.6
Small amount	511	26.0
Moderate amount	158	8.0
Large amount	27	1.4
How much does leaking urine interfere with your daily life?		
Nil (score = 0)	1,246	63.4
Moderate (scores <4)	430	21.9
Severe (scores >5)	288	14.7
Grades of urinary incontinence		
Absent	1,032	52.5
Present	932	47.5
Slight	526	26.8
Moderate	321	16.3
Severe	85	4.3

Table 3 shows that participants’ grades of urinary incontinence differed significantly according to their age group, with the least prevalence among those aged less than 18 years, and the highest prevalence of severe incontinence among those aged 50 years or more (2.6% and 18.2%, respectively; p < 0.001). Grades of urinary incontinence also differed significantly according to participants’ marital status, with those married or widowed having the highest prevalence of severe incontinence (8.5% and 19%, respectively; p < 0.001). Moreover, grades of urinary incontinence differed significantly according to participants’ number of pregnancies, with severe incontinence being highest among those with twice gravidity or three times or more (8.3% and 7.9%, respectively; p = 0.004). However, grades of urinary incontinence did not differ significantly according to their educational status.

**Table 3:** Grades of urinary incontinence according to personal characteristics of participants

	None		Slight		Moderate		Severe		P
Personal characteristics	No.	%	No.	%	No.	%	No.	%	Value
Age groups									
<18 years	238	51.4	129	27.9	84	18.1	12	2.6	
18-29 years	716	54.4	329	25.0	212	16.1	58	4.4	
30-39 years	43	51.2	29	34.5	8	9.5	4	4.8	
40-49 years	25	31.3	33	41.3	15	18.8	7	8.8	
50+ years	10	45.5	6	27.3	2	9.1	4	18.2	<0.001
Educational level									
Primary/intermediate	27	57.4	9	19.1	9	19.1	2	4.3	
Secondary	315	51.8	174	28.6	97	16.0	22	3.6	
University	690	52.7	343	26.2	215	16.4	61	4.7	0.735
Marital status									
Single	924	53.8	450	26.2	281	16.4	63	3.7	
Married	85	42.3	62	30.8	37	18.4	17	8.5	
Widow	7	33.3	9	42.9	1	4.8	4	19.0	
Divorced	16	66.7	5	20.8	2	8.3	1	4.2	<0.001
Number of pregnancies									
0	953	53.7	459	25.9	292	16.5	71	4.0	
1	14	53.8	4	15.4	7	26.9	1	3.8	
2	10	41.7	9	37.5	3	12.5	2	8.3	
3+	55	39.6	54	38.8	19	13.7	11	7.9	0.004

Table 4 shows that grades of urinary incontinence differed significantly according to the presence of associated diabetes mellitus and renal/urinary tract diseases (p = 0.001 for both).

**Table 4:** Grade of urinary incontinence according to participants’ associated comorbidity

	None		Slight		Moderate		Severe		P
Associated comorbidity	No.	%	No.	%	No.	%	No.	%	Value
Diabetes mellitus									
Absent	947	53.9	463	26.4	279	15.9	68	3.9	
Present	85	41.1	63	30.4	42	20.3	17	8.2	0.001
Renal/urinary tract diseases									
Absent	849	54.3	418	26.7	239	15.3	58	3.7	
Present	183	45.8	108	27.0	82	20.5	27	6.8	0.001

## Discussion

The present study revealed that the prevalence of urinary incontinence among participants in the Asir Region was as high as 47.5%. Its frequency was daily among 5.6% of women. Its severity was slight among 26.8%, moderate among 16.3%, and severe among 4.3% of participants. Leaking urine moderately affected the daily life of 21.9% of participants, while it severely affected 14.7%. The amount of involuntary leaking urine was small in 26%, moderate in 8%, and large in 1.4% of participants. These findings are in accordance with those reported by several studies in Saudi Arabia. In Taif City, Almalki et al. [23] reported that the prevalence of urinary incontinence was 34%. While the prevalence was 41.4% in Jeddah, with daily urinary leakage by 17.2% [6]. In Majmaah, the prevalence was 41.7%, with almost half of them leaking small amount, about one-third leaking moderate amount, and only 4.3% leaking a large amount of urine [14] In Riyadh, the prevalence was 42.6%, with a mild impact in 18.6% and a severe impact in 2.8% of the women, while the amounts of involuntary urinary leakage were moderate in 6.9% of participants, and 29.7% experienced urinary leakage once/day [24]. However, international and regional studies reported different prevalence rates for urinary incontinence. Melville et al. [3] found that urinary incontinence among women in Washington, USA was 45%. In Egypt, El-Azab et al. [12] reported that the prevalence of urinary incontinence was as high as 55%, while it was 49.3% in Kuwait [25], but it was lower in Qatar (20.6%) [11]. It is to be noted that there have been widely varied reported estimates of the prevalence and frequency of urinary incontinence due to the lack of sufficient consensus on its definition or types. Moreover, it was hard to compare the results of one study with those of others since the adopted research designs were also different [26]. Since almost half of the Saudi females suffer from urinary incontinence. Hence, it is of paramount importance that it should be identified and appropriately managed. These findings will update healthcare providers as to the current magnitude of this problem. Our study revealed that urinary incontinence imposed a considerable impact on the quality of life of incontinent women and they were less likely to perform routine housekeeping chores and shopping, and were less likely to participate in social events than women with no urinary incontinence [27]. Our study identified several risks and precipitating factors for urinary incontinence among Saudi women, including older age (50 years or more), marital status (married or widowed), and gravidity (twice or more), in addition to being diabetic or having urinary tract diseases. These findings are in accordance with those reported by Al-Badr et al. [6], who reported that risk factors for urinary incontinence among women in Jeddah were older age (postmenopausal), history of multiple births, and those with diabetes mellitus. Similarly, in Egypt, El-Azab et al. [12] identified multiparity and menopause as significant risk factors for urinary incontinence. Swanton and Gormley [28] stated that the etiology for urinary incontinence is multifactorial. Raising awareness of women regarding some modifiable risks may be necessary to reduce the incidence of urinary incontinence, e.g., maintaining physical activity and pelvic floor muscle training, and the identification of the possible favorable impact of systemic estrogens among postmenopausal women. Also, maintaining glucose control can be emphasized during clinical counseling for diabetic patients.

Study limitations and strengths

Few limitations should be considered, such as missing several possible confounders and co-morbidities (e.g., obesity, chronic cough, and constipation), and detailed obstetric history (e.g., age at first pregnancy) or practice of physical exercise, which may increase or modify the list of risk factors. Moreover, this study did not explore the detailed impact of urinary leaks on women’s quality of life (e.g., disruption of daily prayers schedule or sexual life) or motives to seek medical care for such an embarrassing condition. Although this study assessed urinary incontinence by a valid self-reported questionnaire, the diagnosis was not confirmed by clinical examination or other tests. Therefore, the study design may be susceptible to bias since some interviewees may tend to respond as they think is desired or expected by the researchers. Moreover, this study may not be representative of the whole kingdom, since the study sample was taken from a single area.

However, the present study included a big sample size of Saudi women (n = 1,964) and it is the first attempt to shed light on this common public health problem in the Asir Region and to highlight the urgent need for early detection and management of urinary incontinence among females. Moreover, the findings of our study could serve as a source of information for the healthcare system for the management of urinary incontinence among Saudi females, and it will help to educate patients and healthcare professionals on its early detection and management.

## Conclusions

The findings of this study suggest that urinary incontinence is common among Saudi females. Older age, multiparty, and menopause are significant risk factors for urinary incontinence. Therefore, primary prevention of urinary incontinence is recommended by the provision of the necessary health education through mass media about the prevention of urinary incontinence among females by increasing the strength of pelvic floor muscles, particularly after a pregnancy. Further studies should be carried out.
